# Targeting cancer stem cells with polymer nanoparticles for gastrointestinal cancer treatment

**DOI:** 10.1186/s13287-022-03180-9

**Published:** 2022-10-01

**Authors:** Yao Sun, Bo Li, Qian Cao, Tongjun Liu, Jiannan Li

**Affiliations:** 1 Department of General Surgery, The Second Hospital of Jilin University, No. 218 Ziqiang Street, Changchun, 130041 China; 2 Department of Rehabilitation Medicine, China-Japan Union Hospital of Jilin University, Changchun, 130000 China; 3 Department of Education, The Second Hospital of Jilin University, Changchun, 130041 China

**Keywords:** Cancer stem cells, Nanoparticles, Gastrointestinal cancer, Active targeting, Passive targeting

## Abstract

Nanomaterials are developing rapidly in the medical field, bringing new hope for treating various refractory diseases. Among them, polymer nanomaterials, with their excellent properties, have been used to treat various diseases, such as malignant tumors, diabetes, and nervous system diseases. Gastrointestinal cancer is among the cancers with the highest morbidity and mortality worldwide. Cancer stem cells are believed to play an important role in the occurrence and development of tumors. This article summarizes the characteristics of gastrointestinal cancer stem cells and reviews the latest research progress in treating gastrointestinal malignant tumors using polymer nanoparticles to target cancer stem cells. In addition, the review article highlights the potential of polymer nanoparticles in targeting gastrointestinal cancer stem cells.

## Introduction

Gastrointestinal (GI) cancer is among the malignant tumors with the highest morbidity and mortality globally, which mainly include liver cancer, pancreatic cancer, gastric cancer, bowel cancer, and others [1]. Although the current treatment methods for GI cancers are diverse, such as surgery, chemotherapy, radiotherapy, and molecular targeted therapy, the tumor metastasis and recurrence cannot be completely prevented [2]. Therefore, exploring more effective methods to treat GI cancers is necessary.

Cancer stem cells (CSCs) are tumor cells having the characteristics of stem cells and are closely related to tumor occurrence, invasion, drug resistance, and recurrence [3]. CSCs were first found in colorectal cancer [4]. Because of its heterogeneity, drug resistance etc., it was difficult to completely eliminate CSCs using traditional treatment methods [5]. The remaining CSCs can cause tumor recurrence and invasion, and ultimately the death of the patient. Therefore, developing a therapy that can effectively target CSCs will greatly promote the treatment of GI cancers.

**Fig. 1:**
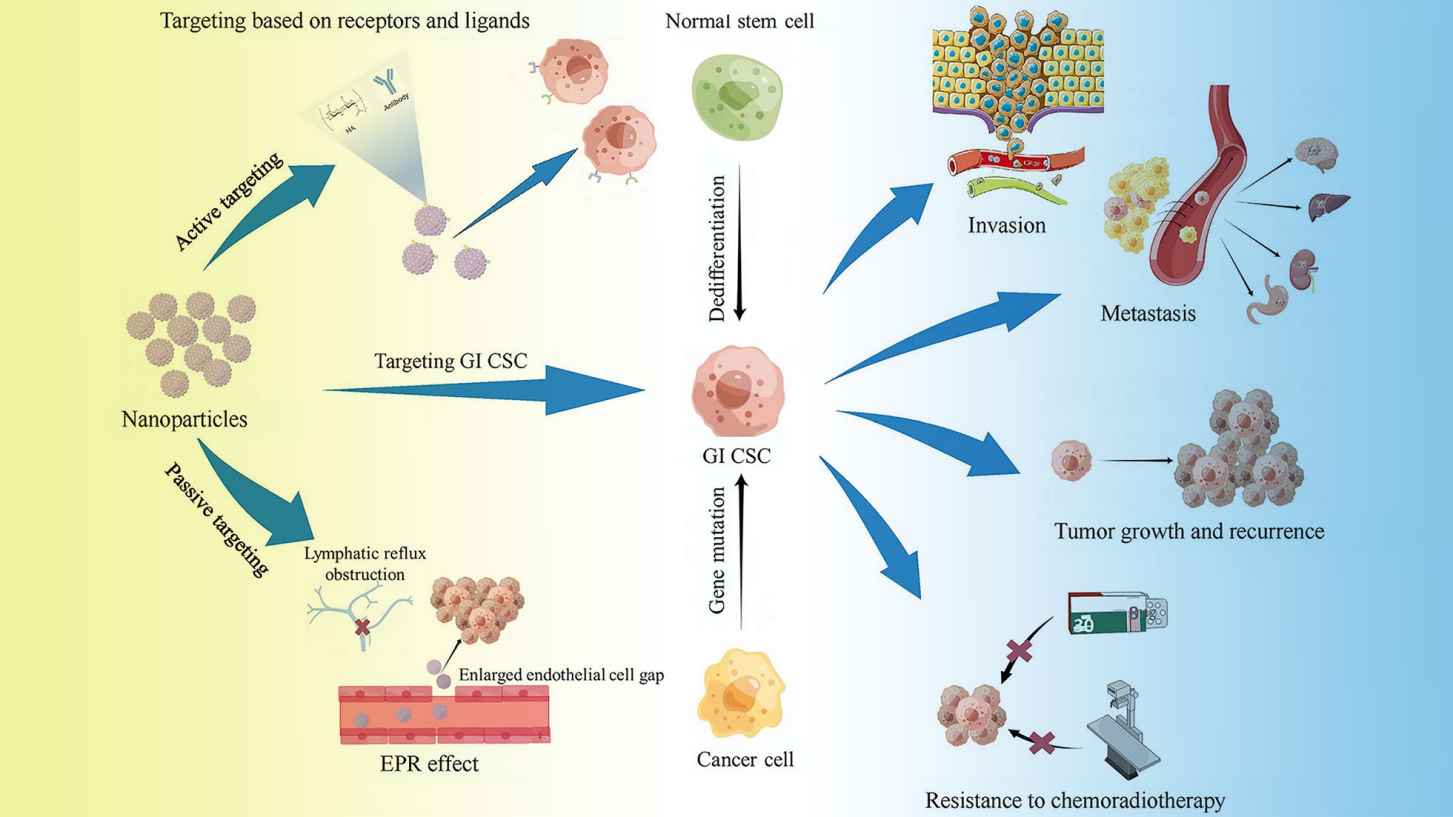
Main ways of targeting CSCs by nanoparticles and the role of CSCs in tumorigenesis development. *EPR effect* enhanced permeability and retention effect; *HA* hyaluronic acid, *GI CSC* gastrointestinal cancer stem cells;

Polymer nanoparticles (PNP) refer to a collection of polymer particles in a size range of 1–1000 nm [6], with diversified structural forms, such as nanocapsules, nanospheres, micelles, and dendrimers [7]. Different forms of PNP have different characteristics. The nanocapsule is a vesicle system, the “cargo” is confined in its water core, and the outside is a polymer shell. Nanospheres include substantial nanoparticles, and the “cargo” is on its surface or wrapped in it [8]. A dendrimer is a three-dimensional macromolecule with a highly branched structure. With strong flexibility, the constituent materials can affect their surface and internal properties [7]. The matrix material of PNP is biodegradable or non-biodegradable but can be safely excreted from the body through the kidneys and other means. Most materials used in polymer nano-research are approved by the FDA, with good biocompatibility [9]. The surface of PNP can be modified with various small molecules such as RNA and protein to make it have a certain targeting or other diverse functions. The PNP can safely transport the loaded drugs to the target site to play a role, avoiding the phagocytosis of the phagocytic system and adverse reactions with other sites. While the systemic side effects are reduced, the drug toxicity increases in the target area [10]. In addition, the PNP drug delivery system can control the release rate of the drug by changing conditions such as pH and magneto-thermal environment to prolong its action time in the target area [11, 12]. Due to the extracellular matrix of solid tumors and cancer-associated fibroblasts (CAFs), some obstacles exist for PNP to reach the intended site. Researchers are trying to overcome the above difficulties by utilizing PNP combined with biological enzymes, regulating CAFs activation, and using physical methods [13, 14]. In recent studies, researchers have fully used the plasticity of the PNP surface and structure to create PNP with various functions and load various drugs into them. Given the excellent properties of PNP, researchers have extensively studied their applications in tumor-targeted therapy. This article summarizes the latest research progress on using PNP to target GI CSCs, analyzes the main difficulties faced by the current research institute, and discusses future research directions. Figure 1 displays the main ways through which nanoparticles target CSCs and the role of CSCs in tumorigenesis development.

## CSCs

### Heterogeneity

There are two theories about the origin of CSCs. One view is that CSCs have the same characteristics as stem cells and, therefore, may be derived from the gene mutations of normal stem cells. Another idea is that they are dedifferentiated from cancer cells, including cancer cell lines and patient tumor tissues [15]. Recent studies have shown that the reprogramming mechanism can convert differentiated tumor cells into CSCs, and even produce CSCs with different characteristics. For example, studies have shown that certain CSCs can exist with mesenchymal-like features or epithelioid-like features and can switch between them dynamically [16, 17]. This process, affected by multiple effects of the internal and external environment of the cell, drives the heterogeneity of CSCs [18]. The heterogeneity greatly impedes its targeted therapy. It is difficult to find an accurate marker to locate CSCs without affecting other normal cells. Ho et al*.* and Zheng et al*.* verified the heterogeneity of liver CSCs using single-cell genomics and tried to determine the relationship between markers and gene expression characteristics of different liver CSCs. This method helps researchers to better understand and determine the heterogeneity and markers of CSCs [19, 20].

### Drug resistance

Many studies have shown that the resistance of tumors to traditional therapies is closely related to CSCs. Researchers have conducted extensive research on the drug resistance of CSCs. However, the exact cause and mechanism are still unclear. It is currently believed that their drug resistance is divided into innate and acquired resistance. Innate drug resistance mainly refers to the characteristics of CSCs themselves, such as drug efflux through the ATP-binding cassette transporter, DNA damage repair mechanisms, and apoptosis evasion mechanisms. Acquired drug resistance mainly refers to the acquisition of drug resistance, the generation of new mutations in the treatment process that affect the treatment target or the proto-oncogene, or the change in the tumor microenvironment during this process. In short, the drug resistance of CSCs involves various mechanisms and requires further research. To solve its drug resistance, it is necessary to start from multiple aspects and apply multiple methods in combination. A single way to control its drug resistance effect is unsatisfactory [15, 21–23].

### Identification of CSCs

Accurate identification of CSCs is the first step in the targeted therapy of CSCs. There are several ways to target CSCs, such as using specific markers, pathways, and tumor microenvironment around CSCs.

In the field of the GI tract, the most widely used CSCs-specific markers are CD44 and CD133 [5]. The former is a transmembrane glycoprotein and plays an important role in tumorigenesis, metastasis, and recurrence. Hyaluronan (HA) and CD44 antibodies are considered important ligands for CD44. The HA-CD44 pathway is closely related to the epithelial–mesenchymal transition (EMT) program, anti-oxidative stress, and epigenetic control. Different CD44 subtypes may be involved in the metastasis and recurrence of different malignant tumors; however, the research on the role of different CD44 subtypes remains incomplete, and further exploration is needed [24]. Similarly, CD133 is also a transmembrane glycoprotein marker closely associated with GI CSCs. The targeting ligand can be a CD133 antibody or RNA ligand [11, 25]. In addition, CD24, CD166, aldehyde dehydrogenase (ALDH), etc., have also been proved to be markers of GI CSCs (Table 1) [26–30]. Although these markers can be used as a basis to identify CSCs, some CSCs populations do not express these markers, and non-CSCs cancer cells may also express them. Therefore, these markers can be adopted to identify subpopulations enriched in CSCs, but may not accurately isolate all CSCs [31].

**Table 1:** Primary markers of GI CSCs

GI cancer	Markers	References
Gastric cancer	CD44, CD133, EpCAM	[27, 31]
Pancreatic cancer	CD133, CD44, CD24, CXCR4, c‐Met, ALDH, ABCG2, EpCAM	[30]
Liver cancer	CD133, CD49, CD90, CD44, CD24	[26]
Colorectal cancer	CD133, CD44, CD24, CD166, EpCAM	[28]

The main reasons for the poor prognosis of GI malignancies are tumor recurrence and metastasis. After standard radiotherapy or chemotherapy, a few surviving CSCs can still lead to tumor recurrence through related pathways. Therefore, it is also essential to specifically target the relevant signaling pathways to alter or block them. Current research shows the main signaling pathways related to GI malignancies: Notch signaling pathway, WNT/-beta-catenin pathway, Hedgehog (Hh) pathway, etc. These pathways play an important role in the occurrence, growth, differentiation, metastasis, invasion, and other processes of CSCs [32]. Although different signal pathways have different roles, they are also inextricably linked. The action on one pathway may influence the other pathway. Acting on multiple pathways at the same time may have a better curative effect. Moreover, CSCs can be dedifferentiated from differentiated tumor cells, so it is critical to specifically inhibit dedifferentiation-related pathways while targeting CSCs themselves.

The specific tumor microenvironment around CSCs provides the basis for passive targeting of nanoparticles, such as pH and enhanced permeability and retention effect (EPR). The researchers achieved passive targeting of tumor areas by tuning the material or composition of the nanoparticles to let them have specific physical or chemical properties. There are also studies combining MRI, photothermal therapy, and other methods with nanoparticles to achieve more diversified diagnosis and treatment methods.

### Culture of CSCs

Studies have shown that when a specific tumor microenvironment is lost, CSCs will have less prominent stem cell characteristics and gradually differentiate into their daughter cells [33]. Therefore, it is essential to establish a medium suitable for CSCs in vitro and cultivate a certain number of CSCs to study the occurrence and development of CSCs and the screening of drugs. The researchers established a culture medium on chitosan membrane to culture colon cancer and liver cancer cells to observe tumor progression and the characteristics of CSCs. The results showed that the chitosan medium could be used to mimic the microenvironment of CSCs and increase the expression of stem cell characteristics-related genes (OCT4 and NANOG) and CSCs markers (CD44, CD24, CD133, CD90, and EpCAM). Chitosan can promote the expression of CSCs population silence regulator (p16 and p21), make CSCs in the G0 phase to prevent their differentiation and decrease their number [34]. In another study, the researchers established a microporous cellulose 3D scaffold culture system that can better simulate the 3D tumor growth microenvironment than the 2D culture system. In addition, cellulose has good biocompatibility and biodegradability. The CSCs cultured on the 3D cellulose scaffold are spherical, making the cells have a better nutritional status, better simulate the living environment and interaction of CSCs, and enrich CD44+/CD133+ cells. Therefore, the structure of the cellulose scaffold and its physical and biological characteristics promote the growth and efficient enrichment of CSCs [35]. In general, two main methods are used to isolate and culture CSCs. In one method, CSCs are selected using special markers on the surfaces of CSCs. However, the CSC surface markers are seemingly inconsistently expressed on CSCs of the same cancer type, which hinders the selection process [36]. In the other method, CSCs are enriched in specific media, such as hanging drops, gyratory rotation and spinner flasks, and ultralow attachment plates. Ultralow attachment plates are more commonly used. Researchers developed a non-adherent 3D culture method, which is simple and low-cost with reusable agar-coated plates, to continuously enrich CSC spheroids [37]. However, some researchers pointed out that common cancer cells were also seemingly enriched in the above method, and CSCs could not be selected completely and accurately. To overcome the above difficulties, the researchers developed a one-single-cell-microencapsulation method to isolate and culture CSCs without surface markers. The results showed that the drug resistance, anti-apoptosis, and multifunctionality of CSCs obtained by this method were significantly better than those obtained by the conventional isolation and culture methods [38].

## Advances in targeting GI CSCs with PNP

In the current study, the methods for targeting GI CSCs with PNP can be roughly divided into active and passive targeting. We list the relevant studies in recent years in Tables 2 and 3 and will discuss them in the following sections.

**Table 2:** Active targeting of CSCs based on PNP

Cancer	Markers	Ligand	Polymers of nanoparticles	Agents	Results	Cell line/Model	References
Gastric cancer	CD44v6	CD44v6 antibody	PEGylated GNSs	NA	CD44v6-GNS nanoprobes actively target gastric cancer cells and inhibit tumor growth under near-infrared laser irradiation	MKN-45 and cancer xenograft mice	[40]
	CD44	HA	Pationic liposomes	Gli1	Gli1 siRNA nanoparticles can specifically block Hh signaling and significantly inhibit CSCs	AGS	[44]
	CD44	HA	PEG-PLGA	METase and 5-Fu	PEG-PLGA NPs coated with HA allow for targeted delivery of 5-Fu and METase to CSCs	NCI-N87	[41]
	CD44	HA	PAMAM	METase	The nanoparticle can disrupt mitochondrial function in CD44-positive gastric CSCs	NCI-N87	[43]
	CD44 and CD133	CD44 and CD133 antibody	PLPN	ATRA	Nanoparticle delivery to two gastric CSCs populations was achieved by diabody conjugation	NCI-N87/MKN-45	[46]
Pancreatic cancer	CD47	CD47 antibody	Iron oleate complex	Gemcitabine	The efficacy of this MMP was demonstrated against CD47-positive pancreatic cancer cells	Panc-1, BxPC-3, and PDX	[59]
	CD133	CD133 antibody	BTiO2	NA	The nanoprobe exhibited high relaxation rate, excellent photothermal efficiency, and targeting ability to pancreatic CSCs in this experiment	SW1990	[63]
	CD44	αCD44 antibody	O^2^LNCs	Paclitaxel	The antitumor efficacy of paclitaxel loaded with αCD44-O^2^ LNCs increased fourfold compared to free paclitaxel	BxPC-3	[60]
	CD44	HA	Co-poly (styrene maleic acid)	3,4-difluorobenzylidene curcumin	Nanomicelles reach tumor sites via enhanced permeability and EPR effect and reach pancreatic CSCs via CD44 receptor-mediated endocytosis	MiaPaCa-2 and AsPC-1	[61]
	CD44	HA	AgNO_3_ -amino acid glutamine	5-FU	Confinement of carboxymethyl inulin significantly alleviates the cytotoxicity of AgNPs and modification of HA improves targeting to CSCs	Panc-1	[62]
	CD326	Anti-human CD326	NaYF4:Yb,Er@NaGdF4	NA	Antibody-modified micelles showed good targeting	BxPc-3 and subcutaneous mouse model	[64]
Hepatic cancer	CD133	Anti-CD133 monoclonal antibody	PEI-FePt	HSV-TK suicide gene	The nanoparticle combination inhibited the growth of hepatic CSCs and induced apoptosis in vitro which was higher than any single intervention	Huh-7	[72]
	CD133	CD133 antibody	PLGA	Paclitaxel	Antibody-modified paclitaxel-loaded nanoparticles can reach target cell populations under passive and active targeting	Huh7	[71]
	CD44	CD44 antibody	Liposomal	Dox	The liposomal nanoparticles were shown to be useful for monitoring and evaluating targeting efficacy and gene therapy by noninvasive molecular imaging	HepG2	[73]
	EpCAM	Anti-EpCAM antibody	NaYF4:Yb,Er	Mitoxantrone	A smart diagnostic and therapeutic micelle based on antibody-conjugated UCNPs was prepared	BEL-7404	[74]
Colorectal cancer	CD133	CD133 antibody	PEG-PCL	SN38	Anti-CD133 antibody-conjugated SN38-loaded nanoparticles can efficiently bind to HCT116 cells	HCT116	[25]
	CD44	HA	PF33-pDNA RRPH	TRAIL	Developed a ternary multifunctional nanoparticle for in vivo gene delivery	HCT116	[82]
	CD133	RNA ligand	Amine‐functionalized dendritic mesoporous silica	Dox	A smart targeted drug delivery system based on PCAD-coated DMSNs was designed, which can control the release of Dox	HT29	[81]
	CD44v6	Fab-CD44v6	Pluronic® F127	Niclosamide	The use of targeted nanoparticles has improved the efficacy of niclosamide in colorectal CSCs	HCT116 and mouse xenograft tumor model	[84]
	CD44/CD168	HA	PEG-PLGA	PTC209	Reversing tumor stemness via orally targeted nanoparticles achieves efficient colon cancer treatment	CT26-luc cells	[85]
	Prominin-1	PROM-1 targeting ligand	Apoferritin and 1-hydroxy-2,5-pyrrolidinedione	Irinotecan	Demonstrated efficacy of irinotecan as a radiosensitizer in a PROM-1-targeted NP formulation	HCT116 and mice ectopic tumor model	[86]

**Table 3:** Passive targeting of CSCs based on PNP

Cancer	Polymers of nanoparticles	Agents	Results	Cell line/model	References
Gastric cancer	PEG-PLGA	SAL and docetaxel	Developed a new strategy to simultaneously target gastric cancer cells and gastric CSCs	MKN-45 /NCI-N87	[49]
	PEG-PLI	miR-34a	A nanoscale stable gene delivery system with low cytotoxicity targeting gastric CSCs was developed	MKN-74	[50]
	PEG-PCL	miR-200c and DOC	MiR-200c and DOC were simultaneously delivered to tumor cells and synergistically inhibited the growth of CSCs and non-CSCs	BGC-823 and cancer xenograft mice	[51]
	PEG-PCL	miR-200c	Nanoparticles loaded with miR-200c can enhance the sensitivity of gastric tumors to radiotherapy by inhibiting CSCs	BGC-823, SGC7901, and MKN-45	[52]
Pancreatic cancer	PEG-b-PLA	SAL	SAL-loaded nanoparticles simultaneously inhibit the proliferation and invasion of cancer cells and CSCs	AsPC-1	[65]
	PLGA	α-mangostin	α-mangostin-PLGA can inhibit the proliferation of pancreatic CSCs and cancer cell lines and the self-renewal capacity of CSCs	AsPC-1, MIA PaCa-2, and PANC-1 cell line	[67]
	PEG-PLGA	Glabrescione B	Nanoparticles prolong the in vivo circulation time of glabrescione B and exhibit specific activity against CSCs	PANC-1	[68]
	PLGA	SAL	SAL-loaded PLGA nanoparticles could be a promising system for the treatment of pancreatic cancer	AsPC-1 and orthotopic pancreatic model	[66]
Liver cancer	PLGA	DS	In combination with copper, DS-PLGA significantly inhibited the liver CSCs population	Huh7	[75]
	PEG-modified LA-SN38	SAL	The nanoparticles promoted apoptosis of liver cancer cells and reduced the proportion of hepatic CSCs	Human HCC and CDX	[76]
	Chitosan	DDC	Based on pH differences, the nanoparticles can be used to selectively target cancer cells with minimal impact on normal tissues	Black mouse C57	[77]
	Nanodiamonds	Epirubicin	Nanodiamond-mediated epirubicin delivery may serve as an effective approach to overcome chemoresistance in CSCs	Murine hepatoblastoma tumor model	[79]
	DMPC and C12(EO)23	NA	HLs have inhibitory effects on the growth of hepatic CSCs populations in vitro	HepG2	[78]
Colorectal cancer	Lipid	SN38 and SAL	Nanoparticles loaded with SN38 and SAL are effective against therapy-resistant dormant CSCs and cancer cells	HCT116	[92]
	Nanocrystals	SAL	SAL nanocrystals show higher cellular uptake efficiency and tumor accumulation compared to free SAL	HCT116 and HT29	[93]
	Cubic iron oxide and thermoresponsive polymer	Dox	A smart nanoplatform that combines both thermal and chemotherapy was produced	Nude mouse xenograft model	[12]
	PLGA	α-mangostin	Mang-NPs can inhibit cancer cell growth, EMT, and the number of CSCs by inhibiting the Notch pathway	HCT116/HT29	[94]

### Gastric cancer

Gastric cancer is the fourth leading cause of cancer-related death worldwide [39]. Because its recurrence and metastasis are difficult to be completely eliminated by traditional treatment methods. Gastric CSCs are considered to be closely related to the occurrence, recurrence, and metastasis of gastric cancer. Researchers are using nanoparticles to target gastric CSCs for more sensitive and specific treatments.

At present, CD44 is the most widely adopted marker in the targeted research on gastric CSCs, and CD44v6 is a variant of it with better specificity in Gastric cancer. In a study, the authors prepared CD44v6 monoclonal antibody-conjugated polyethylene glycol (PEG)-modified gold nanostars (GNS) nanoprobes (CD44v6-GNS-PEG). The results showed that CD44v6-GNS-PEG could target gastric CSCs with good stability and biocompatibility, the binding rate of the CD44v6-GNS-PEG group was 2.5 times higher than that of the control group. In addition, the tumor growth of tumor-bearing mice was significantly inhibited under near-infrared laser irradiation. Because of the high thermal conversion efficiency and photothermal ablation ability of CD44v6-GNS-PEG, it overcomes the resistance of CSCs to ordinary photothermal therapy to a certain extent. It showed great potential for targeted imaging and photothermal therapy of gastric CSCs [40]. In addition to CD44 antibodies, HA is also an important targeting ligand for CD44. The researchers coupled HA on the surface of PEG-poly (lactic-co-glycolic acid) (PLGA) nanoparticles and loaded L-methionine-deamino-γ-mercaptomethane lyase (METase) and 5-fluorouracil (5-Fu) into the nanoparticles. Most cancer cells require high levels of methionine (MET) for growth. Overexpression of METase inhibited CSC proliferation by down-regulating MET levels, increasing the levels of Cyc-C, reactive oxygen species (ROS) involved in mitochondrial function, and apoptosis-related proteins c-caspase 3. METase has a specific inhibitory effect on cancer cells, without an obvious effect on normal cells. The results showed that the combination significantly reduced the level of MET, and the HA-coated METase nanoparticles were more efficient in decomposing MET [41]. In addition to PEG-PLGA, polyamidoamine (PAMAM) is a highly branched dendritic macromolecule with a cavity structure, high transfection efficiency, and low toxicity and is widely used for drug delivery [42]. In a study, the authors modified PAMAM with HA to deliver METase to CD44-positive gastric CSCs. The modification of HA reduces the intrinsic toxicity of PAMAM and improves the interaction efficiency of the nanoparticles with the carried METase. Furthermore, they also utilized AuNPs to help PAMAM retain the 3D spherical shape of dendrimers and improve gene transfection efficiency. The experimental results showed that the combination inhibited the proliferation of tumor cells and reduced the number of CD44-positive CSCs [43]. In another study, the researchers loaded glioma-associated oncogene homolog 1 (Gli1) small interfering RNA (siRNA) into HA-modified di-stearoyl-phosphatidyl-ethanolamine (DSPE) nanoparticles. The Gli1 is a downstream protein of the Hh signaling pathway. Therefore, delivering siRNA to CSCs to inhibit Gli1 expression may become a new strategy for cancer therapy. This study demonstrated that nanoparticles were selectively directed to CD44^+^ gastric CSCs and then internalized through CD44 receptor-mediated endocytosis. The nanoparticles were encapsulated in endosomes or lysosomes and subsequently escaped from the endosomes or lysosomes (rather than being degraded or leaking out of the cell) to act in the cytoplasm. This phenomenon may be attributed to the membrane-disrupting properties of cationic carriers [44]. The targeting function effectiveness of HA, targeting ligand of CD44, has been proven in the above experiments. In addition, the presence of HA, with excellent biodegradability, non-toxicity, and non-immunogenicity, provides a hydrophilic protective layer for the nanoparticles, prolonging their time in the blood circulation. These properties offer a broad field for HA to target CSCs.

Among the targeted studies on gastric CSCs, the current studies target CD44^+^ cells alone. However, CSCs are mostly composed of multiple populations that have different markers, so the targeted therapy on multiple markers simultaneously may achieve better therapeutic effects [45]. In the study of Chen et al*.*, a lipid-encapsulated PNP was designed, and CD44 and CD133 antibodies were simultaneously conjugated to it to prepare CD44/CD133-ATRA-PLPN (poly(lactide-co-glycolide)-lecithin-PEG). All-trans retinoic acid (ATRA) has a good therapeutic effect on various CSCs; however, its solubility and bioavailability are poor, which can be overcome by loading ATRA into lipid-encapsulated PNP. The results of this study demonstrate that CD44/CD133-ATRA-PLPN can be efficiently and specifically delivered to CD44-positive or CD133-positive gastric CSCs compared with single-targeted and non-targeted nanoparticles. In contrast, ATRA-PLPN without antibody conjugation cannot target any cell population. This study also shows the enhanced targeting of diabody-conjugated PNP, validating its effectiveness. However, as a small part of the tumor population, gastric CSCs are not used to investigate the optical imaging and in vivo distribution of CD44/CD133-ATRA-PLPN [46]. Two antibodies are coupled on one nanoparticle in this experiment, enabling efficient targeting of different antigen-positive populations. Perhaps when the same population has multiple effective antigens, we can design nanoparticles that target multiple antigens simultaneously to achieve “multiple” targeting. Multiplex targeting is extremely promising in targeting CSCs, but studies in the field of GI CSCs are still scarce. Some researchers designed a dual-targeted iron oxide nanoparticle (IONP) carrying Dox. They coupled Wnt receptor binding peptide and urokinase plasminogen activator receptor targeting ATF_24_ peptides to this IONP to specifically inhibit the Wnt/β-catenin pathway and CSC populations. The inhibitory effect of the dual-targeted IONP was greater than that of a single-targeted or untargeted IONP [47]. The design idea in this study is significantly enlightening for targeting GI CSCs and can be extended to explore the therapeutic effects on GI CSCs.

In addition to active targeting, researchers have also tried to target gastric CSCs only by passive targeting. Salinomycin (SAL) is considered to have a strong inhibitory effect on various CSCs; however, its poor solubility limits its application. The researchers suggested that SAL constrains gastric tumor growth by inhibiting the Wnt signaling pathway in CSCs. Strong positive correlations were among the level of Wnt expression, gastric CSC markers, and the immunohistochemical expression of CD44. Wnt increased the percentage of the S phase of the cell cycle by accumulating cyclins D1 and D2 to stimulate cell proliferation [48]. In a study, the authors loaded SAL and the anticancer drug docetaxel into PEG-PLGA nanoparticles. The PLGA is highly biocompatible and safe, and PEG modification prolongs the systemic circulation time of nanoparticles and thus enhances the passive targeting of nanoparticles. Since loading two drugs into the same nanoparticle is difficult to synergize their drug release rates and the manufacturing process is complicated, the two drugs are simultaneously loaded into two separate nanoparticles. In this study, we validated the therapeutic effects of nanoparticles loaded with both SAL and traditional antitumor drugs in MKN-45 and NCI-N87 human GC cell lines. The results showed that this combination effectively inhibited tumor growth, and SAL significantly reduced the number of CSCs in the tumor [49]. Certain RNA molecules can also inhibit the growth of gastric CSCs or increase their sensitivity to chemoradiotherapy. However, they are easily affected by nucleases or polyanionic nature, impeding their in vivo transport. The above difficulties can be solved by transporting nanoparticles. As a negative regulator of CD44 expression, miR-34a is frequently under-expressed in tumor tissue. The CD44 signaling pathway is important in the initiation, proliferation, and migration of CSCs. Therefore, introducing miR-34a mimics (or miR-34a-containing vectors) can reduce CSCs. The researchers encapsulated miR-34a into nanoscale PEGylated liposomal vesicles to target CD44-positive cancer cells. The transport of gene molecules to target cells is realized, and the in vivo transport efficiency and therapeutic effect of RNA molecules are improved [50]. Liu et al*.* and Cui et al*.* enhanced the sensitivity of gastric tumor cells to chemotherapeutic drugs and radiotherapy and inhibited the growth of CSCs by delivering miR-200c from nanoparticles to gastric tumor cells [51, 52]. Liposomes, which are artificial nanovesicles with phospholipid membranes, are one of the most promising drug delivery systems. They are easy to prepare and can circulate in vivo for a long time; however, their immunocompatibility and targeting effects need to be improved [53]. In addition to artificial nanovesicles, cell-secreted extracellular vesicles are also a research hotspot in drug delivery systems in recent years. They inherit many characteristics of parental cells and have good biocompatibility. Moreover, they can have more excellent properties through the modification of surfaces, structures, and contents [54]. Tumor-secreted extracellular vesicles are important in promoting tumor metastasis, angiogenesis, and the transformation of tumor cells into CSCs. Based on tumor-secreted vesicles, nano-drug delivery vehicles targeting CSCs were fabricated to effectively target and deliver drugs to CSCs [55]. In general, nanoparticles loaded with drugs or molecules targeting gastric CSCs can achieve targeted transport to gastric tumor cells under the action of EPR. However, some studies have also pointed out that the EPR effect is heterogeneous among different tumors, species, and stages of tumor development. Thus, the targeting efficiency of nanoparticles relying on the EPR effect to achieve passive targeting is different [56]. Therefore, it is necessary to further accumulate various experimental data on the EPR effect to lay a foundation for the clinical application of nanomedicines.

### Pancreatic cancer

Pancreatic cancer is a malignant tumor that severely threatens the lives of people worldwide. Most patients have a short survival time and a poor prognosis. Since it has no special symptoms, it has mostly metastasized to the surrounding blood vessels and digestive tract when diagnosed. In addition, the complexity of its nearby anatomical structure causes a low chance of surgical treatment. Therefore, it is critical to developing an effective targeted treatment plan for pancreatic cancer [57, 58].

At present, the nanoparticles designed by researchers for pancreatic CSCs can target pancreatic CSCs by actively targeting CD47, CD133, αCD44, and CD326. In a study, the authors covalently attached the CD47 antibody to multifunctional iron oxide magnetic nanoparticles and mixed the chemotherapeutic drug gemcitabine into them. This study was conducted in Panc-1 and BxPC-3 cell lines and patient-derived xenograft models (PDX), and the results showed good therapeutic effects with good stability and targeting of the combination in in vivo and in vitro studies. However, further improvement is still needed. For example, it does not cover the surface of the nanoparticles with polymers or other materials to improve their stability in the circulatory system [59]. Researchers are also constantly developing and exploring new nanomaterials to improve drug delivery efficiency. In another study, the researchers designed an αCD44 antibody-modified olive oil liquid nanocapsules (O^2^LNCs) drug delivery vehicle for the targeted delivery of paclitaxel to pancreatic CSCs. The results showed that the antitumor effect of paclitaxel delivered by αCD44-O^2^LNCs was fourfold higher than that of free paclitaxel. In fluorescence imaging, O^2^LNC that was not modified by the antibody was evenly distributed in mice, while αCD44-O^2^LNC was distributed in mice but was significantly accumulated in the tumor area. This structure relies on its biocompatibility and biodegradability, with olive oil as its main ingredient. Then, hydrophobic drugs were encapsulated in its core–shell structure, and specific antibodies were coupled to its surface in a covalently modified manner to achieve targeted trafficking of pancreatic CSCs [60]. Both groups of experiments demonstrate that antibody-modified nanoparticles have improved the targeting ability to pancreatic CSCs, and the application of polymer materials can greatly enhance the drug delivery efficiency.

In addition to the CD44 antibody, the researchers designed a HA conjugate of co-poly (styrene-maleic acid) (HA-SMA) utilizing the targeting of HA to CD44. The relatively simple coupling of SMA anhydrides allows the modification of sugar residues on HA polymers for structural versatility and facilitates the self-assembly and encapsulation of hydrophobic drugs. HA-SMA forms nanomicelles (HA-SMA-CDF) with 3,4-difluorobenzylidene curcumin (CDF). HA-SMA-CDF reaches the tumor site based on a strong permeability and retention EPR effect and reaches CD44 overexpressing pancreatic CSCs through an active targeting mechanism (CD44 receptor-mediated endocytosis). HA-SMA-CDF down-regulates the expression of NF-κB in CD44^+^ cells, and inhibiting the expression of NF-κB target genes may inhibit the proliferation and invasion of pancreatic CSCs [61]. In the experiments of Joshi et al., HA-conjugated silver nanoparticles with graphene quantum dots were also successfully and specifically targeted to pancreatic CSCs. The toxicity of silver nanoparticles was reduced by linking carboxymethyl inulin to the nanoparticles [62]. From the above studies, we can find that HA can be adopted as a CD44 ligand to target pancreatic CSCs. The existence of functional groups provides the possibility to fabricate multifunctional nanoparticles.

The early and accurate diagnosis of pancreatic cancer is a major bottleneck in the diagnosis and treatment of pancreatic cancer, which may be improved by combing nanotechnology and imaging. Researchers applied MRI to nanoparticle-targeted therapy. Wang et al. loaded Gd-DOTA and CD133 monoclonal antibodies on black TiO2 (BTiO2) nanoparticles to achieve targeted photothermal therapy of pancreatic CSCs under the guidance of MRI. The BTiO2 is a photothermal agent with high photothermal conversion efficiency and low toxicity. In recent years, it has been widely used as a drug transport carrier. The results showed that pancreatic CSCs with a high expression of CD133 have a strong uptake of CD133 monoclonal antibody-coupled nanoprobes and can enhance the imaging effect of MRI on CSCs and improve the photothermal ablation efficiency of pancreatic CSCs [63]. In addition, CD326 is another promising target for pancreatic CSCs. The researchers achieved dual-mode imaging of fluorescence and magnetic resonance by coupling the CD326 antibody to gadolinium ion-doped upconversion nanoparticles (UCNPs). With the help of CD326 antibody conjugation, UCNPs achieved good active targeting ability [64]. The combination of MR and nanoparticles provides the possibility of early diagnosis of pancreatic cancer and can be applied to imaging-guided targeted therapy. There are still few related studies and applications which are worthy of further research.

Studies have shown that some molecules themselves, such as SAL, glabrescione B, and α-mangostin, also have a targeted inhibitory effect on pancreatic CSCs; however, their applications are limited because of their poor water solubility, unsatisfactory stability, or short half-life. The application of nanomaterials technology can solve these problems and increase their roles in targeting tumor stem cells in pancreatic cancer treatment. In a study, the authors delivered SAL loaded in PEG-b-PLA polymeric micelles to drug-resistant pancreatic cancer cells. Better penetration effect and EPR effect can be achieved when the nanoparticle size is less than 200 nm. They prepared PEG-b-PLA polymer micelles with a diameter of 154.5 ± 10.6 nm. The results of this study showed that compared with free SAL, PEG-b-PLA-loaded SAL exhibited better pharmacokinetics and inhibitory effect on the proliferation and invasion of cancer cells and CSCs [65]. The group further investigated the anti-pancreatic tumor properties of SAL-loaded nanoparticles by loading them into more hydrophobic PLGA nanoparticles in a follow-up study. The results suggest that SAL may affect pancreatic tumor cells by targeting the mesenchymal–epithelial transition. However, the exact mechanism remains unclear [66]. In the study of Verma et al*.*, PLGA nanoparticles were used to encapsulate α-mangostin, which improves its bioavailability and accumulation in target organs. In this experiment, α-mangostin-PLGA was well absorbed by CSCs and cancer cells. It inhibited the occurrence, growth, and metastasis of CSCs by suppressing the expression of EMT transcription factors, the Hh pathway and its downstream targets, and constraining pluripotency maintenance factors [67]. Similarly, the researchers also achieved a targeted inhibition effect of the Hh pathway in vivo and in vitro by encapsulating glabrescione B in nanocapsules. Glabrescione B bound to the Hh regulator Gli1 and exerted its activity by interfering with Gli-DNA interaction. In addition, it has been proved that glabrescione B encapsulated in nanocapsules has the same activity as the free drug and less cytotoxicity to non-CSCs cells [68]. Several of the above studies have shown that the application of PNP improves the bioavailability and pharmacokinetics of related molecules compared with free molecules and does not affect their antitumor activity.

### Liver cancer

Liver cancer is the third leading cause of cancer-related deaths. Current research shows that the existence of CSCs causes high invasiveness, recurrence rate, and resistance to existing treatments of liver cancer [69, 70]. Therefore, researchers are trying to create nanoparticles that target liver CSCs to treat liver cancer.

Like other GI tumors, the most widely studied specific markers in the field of liver CSCs are CD133 and CD24. In a study, the authors conjugated the CD133 antibody to PLGA nanoparticles and then loaded paclitaxel into the nanoparticles. Under the dual action of active and passive targeting, the nanoparticles successfully and selectively eliminated CD133-positive cancer cell populations [71]. With the development of radiotherapy and gene therapy, some researchers have also applied it to target liver CSCs. Lin et al*.* used iron–platinum nanoparticles (FePt-NPs) as a carrier and conjugated CD133 antibody to it and prepared a strategy combining HSV-TK suicide gene, ^131^I nuclide irradiation, and magnetic fluid hyperthermia. This combined strategy successfully transfected the suicide gene into target cells and induced its expression, achieving the “dual effect” of radiation therapy and gene therapy. The mechanism may be related to the down-regulation of VEGF and CD44 protein expression, thereby inhibiting tumor angiogenesis and the proliferation and invasion of CSCs [72]. This study provides a novel non-viral gene carrier and magneto-sensing mediator and demonstrates that the presence of the CD133 antibody facilitates the targeting of therapy.

Monitoring the regression and progression of CSCs is essential in the diagnosis and treatment of HCC. The researchers conjugated CD44 antibody and a trifusion plasmid consisting of monomeric red fluorescence protein, renilla luciferase, and truncated herpes simplex virus thymidine kinase reporter genes to liposome nanoparticles. Tumor growth and targeting of liposome nanoparticles are monitored and tracked using optical bioluminescence imaging and Rluc imaging [73]. In addition to the above-mentioned two specific markers, EpCAM is also considered to be an important marker of hepatic CSCs. In a study, the authors designed an EpCAM antibody-conjugated upconversion nanomicelle for synergetic chemotherapy and photodynamic therapy guided by dual-modality imaging with magnetic resonance/upconversion luminescence and then loaded mitoxantrone into it. Photodynamic therapy induces ROS production in target cells, destructing tumor cells and blood vessels, and is an important palliative and noninvasive modality for the minimally invasive treatment of cancer. The results showed that this combination therapy achieved a significant synergistic antitumor effect and imaging effect. The presence of the EpCAM antibody greatly enhanced the uptake of nanomicelles by BEL-7404 cells [74]. In general, the targeting of hepatic CSCs by nanoparticles loaded with antitumor drugs, genes, and specific therapies can be improved using specific antibodies. At the same time, designing suitable imaging methods to monitor and evaluate the targeting efficacy is also necessary before relevant research can be translated into clinical practice.

Some drugs have specific inhibitory effects on hepatic CSCs, such as disulfiram (DS) and SAL. However, because of its poor water solubility or short half-life, the in vivo administration effect is unsatisfactory, which may be overcome using nanoparticle carriers. The researchers encapsulated DS in PLGA to prolong the half-life and anticancer effect of DS in vivo. The DS is an anti-alcoholic drug that has been shown to be cytotoxic to various cancer cells. More critically, DS specifically inhibited the activity of ALDH, a functional marker of CSCs. The results of this study showed that DS-PLGA showed a strong inhibitory effect on ALDH and CD133-positive CSCs population in both hypoxia-induced and strong synergy with 5-FU and Sorafenib. A synergistic inhibitory effect on liver cancer cells and liver CSCs was achieved. Furthermore, the combination inhibited the hypoxia-induced EMT process closely related to CSC transfer [75]. In another study, the researchers loaded SAL into PEG-modified LA-SN38 (linoleic acid-modified 7-ethyl-10-hydroxycamptothecin) to construct a nano-prodrug. 7-ethyl-10-hydroxycamptothecin is converted from camptothecin, which induces apoptosis by inhibiting the activity of the ribozyme topoisomerase I. The ribozyme topoisomerase I is vital in DNA replication. However, its application is limited due to its poor solubility. The modification of linoleic enables it to be amphiphilic and induce self-assembly in an aqueous solution. Modification of polyethylene glycol can enhance the in vivo stability and half-life of nano-prodrug and reduce systemic toxicity. The results showed that SAL loading increased the sensitivity of liver cancer cells to SN38, and a combined administration was more effective than a single administration. However, SAL played a major role in inhibiting CSCs, and SN38 had a little synergistic inhibitory effect. The researchers suggest that this may be related to the resistance of CSCs to the broad-spectrum antitumor activity of SN38 that inhibits DNA synthesis [76]. The nano-co-delivery system proposed in this study represents a new exploration for the synergistic drug delivery system targeting CSCs and tumor cells. In addition, nanoparticles enable the related drugs to reach the target site with higher “efficiency,” which may be related to the EPR effect.

In addition to traditional PEG and PLGA, researchers are constantly exploring new nanomaterials for better targeting. In the study of Abu-Serie et al*.*, a nanoparticle made of chitosan was designed, which was then coated with negatively charged albumin and loaded with diethyldithiocarbamate (DDC). The DDC is considered an inhibitor of ALDH, a key enzyme affecting the survival of CSCs. The results showed that albumin-coated nanoparticles could release DDC in a weakly acidic environment (tumor microenvironment); however, the release amount is less in a neutral environment. The experiments showed that the growth of 89.7% CD133-positive CSCs was inhibited, which may be related to the suppression of NF-κB expression and ALDH activity by DDC [77]. In another study, nanoscale hybrid liposome particles (HLs) were designed, consisting of L-a-dimyristoylphosphatidylcholine and polyoxyethylene (23) dodecyl ether (DMPC and C12(EO)23), they can inhibit the growth of the CSC population by transmitting apoptotic signals through caspases-9, -3, and -8. The results showed that HLs selectively accumulated in the cell membrane of CSCs in a dose-dependent manner. The specific mechanism remains unclear but may be related to the difference in membrane fluidity between CSCs and differentiated cancer cells [78]. In another study, the researchers used epirubicin-loaded nanodiamonds to target liver CSCs in a murine hepatoblastoma tumor model. The drug resistance of CSCs was caused by the recognition and exclusion of epirubicin by ABC transporters on CSCs. Researchers tried to overcome these problems with ABC transporter-specific inhibitors, but the effects were poor. Nano-drug delivery systems have improved the therapeutic efficacy of epirubicin. Nanodiamonds combine with epirubicin through physical adsorption to form a nanodiamond–epirubicin complex (EPND). The ENPD relies on its unique size and surface charge characteristics to passively target tumor regions, increasing the concentration and residence time of antitumor drugs in specific regions [79]. The above groups of studies can target the liver CSCs region to a certain degree using the pH of the tumor microenvironment, the membrane fluidity of CSCs, and the charge characteristics. In general, it is feasible to use the characteristics of liver CSCs and their surrounding environment to fabricate nanoparticles with corresponding “preferences” to target CSCs. In addition, these “preferred” nanoparticles can target liver CSCs, and if the corresponding ligands are attached to them through further research, the targeting ability of these nanoparticles may become stronger.

### Colorectal cancer

The incidence of colorectal cancer is about 9% of all cancers, and surgery is the main treatment method; however, the recurrence rate after surgery is still high. In addition, many patients cannot undergo surgical treatment because of the late stage of the tumor, and thus only palliative radiotherapy and chemotherapy can be taken [80]. Therefore, the treatment of colorectal cancer by targeting CSCs, which are closely related to the occurrence, metastasis, and recurrence of colorectal cancer, has become one of the hot research directions.

In studies using nanoparticles to actively target colorectal CSCs, CD133 and CD44 were the most important targeting markers. In a study, the authors designed a nanocarrier based on PEG-Polycaprolactone (PCL) to load SN38 and conjugate CD133 antibody to its surface to achieve targeted clearance of CD133-positive cells. The results demonstrated that CD133 antibody-conjugated SN38-PEG-PCL nanoparticles could be targeted and safely delivered to colorectal CSCs [25]. Coating other materials on the surface of nanoparticles can give them more functionality. Alibolandi et al*.* prepared a dextran-coated dendritic silica nanoparticle loaded with doxorubicin (Dox) and coupled a specific RNA ligand against CD133 on the surface of the nanoparticle to achieve specificity for the colorectal CSCs target. Dextran is a hydrophilic polysaccharide with good biocompatibility and biodegradability. In addition, some studies have shown that the colloidal stability of Dextran-coated nanoparticles is higher than that of PEG-coated nanoparticles, and they are not easy to adsorb non-specific proteins [81]. The results of this study show that the drug release efficiency of the nano-drug delivery system is closely related to the pH value, and the “cargo” release is accelerated when the pH is 5.4. This may be closely related to the carboxylic acid groups and amine groups on the silica surface of Dextran. In vitro cytotoxicity experiments confirmed that the conjugation of CD133-specific RNA ligand aptamers significantly enhanced the uptake of nanoparticles by CSCs [11]. The pH-triggered drug delivery system designed in this study can reduce the drug wastage in the human bloodstream, and once it reaches the target site in the tumor microenvironment, it can enter the target cell through endocytosis. Since the pH in the endosome is about 5.0, the release efficiency of the drug can be promoted. In this study, a more intelligent targeted drug delivery method was designed, which reduced the loss of drugs during the delivery process, increased the targeting of drug delivery, and provided a new idea for the specific treatment of CSCs.

The CD44 is also a popular marker molecule in the field of colorectal CSCs. In a study, the authors prepared a multifunctional nucleus-targeted nanoparticle for targeted therapy of colorectal CSCs by loading tumor necrosis factor-related apoptosis-inducing ligand (TRAIL). Tumor necrosis factor-related apoptosis-inducing ligand induces apoptosis in a tumor by binding to death receptors 4 and 5 and delivering intracellular death signals. This nanoparticle consists of a targeting core (PF33/pDNA) with extremely high transfection efficiency and a negatively charged capsid-like shell (RGD-R8-PEG-HA) with multi-level targeting activity. The HA was coupled to nanoparticles for targeting CD44-positive CSCs and can be degraded under the action of HA enzyme in tumor cells, thereby promoting the release of load. The RGD peptide in RGD-R8 (Cys-RRRRRRRR-c(RGDfK)) can act as a specific ligand for the integrin αvβ3 receptor, and R8 can mediate the penetration of nanoparticles into the interior of tumor spheres. Integrin αvβ3 is thought to play an important role in tumor angiogenesis. The results obtained by the researchers in the xenograft tumor model and HCT116 cell line showed that this nanoparticle with multiple targeting functions could deliver TRAIL to the tumor area, inhibit tumor cell growth, promote apoptosis, and reduce the proportion of CSCs. This multi-site targeted gene delivery system provides a new idea for the targeted therapy of colorectal CSCs [82]. In another study, the researchers prepared a polymeric micelle loaded with niclosamide, which was then modified with antibody fragments against CD44v6 to target CD44v6-expressing colorectal CSCs. Studies have shown that niclosamide inhibits mTORC1 (a key regulator of autophagy) by disrupting the cytoplasmic pH balance, thereby inactivating the Wnt signaling pathway [83]. The results showed that niclosamide was successfully delivered to CD44v6 overexpressed tumor cells in the mouse xenograft tumor model and HCT116 cell line [84]. Chen et al*.* and Zhang et al*.* also achieved selective targeting of CSCs through specific ligand-conjugated nanoparticles [85, 86]. From the above studies, we can find that the coupling of ligands can significantly improve the targeting of nanoparticles to colorectal CSCs. In addition, the further modification of the nanoparticles enables them to have more complex functions, thereby improving the transport and release efficiency of “cargo.”

Antibody-based protein therapy is a promising approach in cancer treatment due to its high selectivity and specificity. However, since most antibodies cannot penetrate cell membranes, they usually only act on extracellular targets [87]. The development of nanotechnology has opened up new possibilities for the intracellular delivery of antibodies. In a study, the authors prepared an amphiphilic polymer-based nanomicellar delivery system for delivering specific antibodies against structural maintenance of chromosome protein 2 (SMC2) into CSCs. Its combination therapy strategy with traditional anticancer drugs paclitaxel and 5-Fu was also discussed. The results of this study showed that the specific antibodies against SMC2 were successfully released intracellularly, and the efficacy of the two anticancer drugs carried was higher than that of those in free forms, showing higher cytotoxicity to CSCs [88]. The SMC2 is closely related to sister chromatid segregation and regulation of interphase during cell division and has been shown to be overexpressed in diversified tumors, including colorectal, gastric, and pancreatic cancers [89, 90]. However, there are still few studies on the use of nanoparticles to achieve SMC2 delivery, which is worthy of further studies.

The passive targeting of colorectal CSCs with nanoparticles has also been extensively explored. Sal is also thought to selectively induce apoptosis of colorectal CSCs [91]. In a study, the authors encapsulated SN38 and SAL in lipid nanocapsules to utilize the EPR effect of the tumor region to achieve passive targeting of the region. The results showed that SN38 mainly acts on the proliferation of cancer cells, and SAL acts on CSCs. The two drugs were encapsulated in nanocapsules. Thus, they have lower systemic toxicity and better targeting and synergistic effects [92]. In addition to lipid nanoparticles, the development of drug nano-crystallization technology provides new ideas for improving the drug delivery efficiency of SAL. In another study, the researchers prepared SAL nanocrystals with good size distribution, stability, and water solubility. It was confirmed under fluorescence imaging that SAL nanocrystals both in vivo and in vitro showed better tumor area accumulation and cellular uptake rates than free SAL. In addition, they showed better anticancer effects in an orally administered mouse cancer model. Oral administration has great advantages in treating GI tumors because of its simplicity, noninvasiveness, and high intestinal drug concentration. The SAL nanocrystals fabricated in this study showed higher drug concentrations in the colonic region and lower systemic side effects because of their unique surface chemistry and size [93]. Similar to SAL, α-mangostin also has limited application in anticancer therapy in vivo since its water solubility and stability are poor, with low accumulation in target organs. Boinpelly et al*.* prepared PLGA nanoparticles containing α-mangosteen (Mang-NPs). In this study, α-mangostin was loaded into PLGA nanoparticles, which overcame the above shortcomings to a certain extent. The results showed that Mang-NPs inhibited the self-renewal of CSCs by inactivating the Notch signaling pathway, down-regulating the expression of stem cell markers and multifunctional maintenance factors. It also inhibits cell viability and EMT processes, induces apoptosis in cancer cells, and avoids affecting normal colorectal epithelial cells [94].

Researchers have explored multiple strategies to target colorectal CSCs with nanoparticles. However, most methods still cannot completely eliminate CSCs in tumor tissue. There may be a mutual transformation between some CSCs and tumor cells. Eliminating both of them is critical; however, it also adds complexity to the study. Therefore, some researchers try to reduce the difficulty of eradicating CSCs by promoting the transformation of CSCs into differentiated tumor cells. In a study, the researchers prepared magnetic nanoparticles composed of cubic iron oxide nanomaterials and coated the nanoparticles with a thermally responsive polymer. Such thermally responsive magnetic nanoparticles were used to deliver Dox to tumor areas in the nude mouse xenograft model. Under the action of an alternating magnetic field, heat is generated to excite the magnetic nanoparticles to release Dox. The magnetic nanoparticles had a cytotoxic effect on differentiated tumor cells under an alternating magnetic field. At the same time, heat stress caused CSCs to exit the dormant state and reenter the differentiated state. During this process, Dox in the nanoparticles was released under thermal stimulation and successfully internalized by CSCs, resulting in the death of CSCs [12]. This study eradicated CSCs by triggering their differentiation and avoided tumor recurrence. This provides us with a new way of thinking, and we can indirectly eliminate them by promoting the differentiation of CSCs and then eradicate tumors.

## Discussion and perspective

The CSCs have become a huge obstacle to anticancer therapy because of their characteristics and are also important target cells for many anticancer treatments. In recent studies, targeting GI CSCs with polymeric nanoparticles has emerged as a promising approach. The advantages and the problems to be solved are summarized as follows:

1. With the development of material science, PNP with increasing diverse materials, shapes, and functions has been developed. However, the available polymer materials in this field are still relatively simple, most of which are based on PEG, PLGA, etc. In addition, different synthetic processes, structural shapes, and ligands coupled to the surface of the same material will lead to nanoparticles with different functions. Therefore, it requires material and medical researchers to jointly develop more attractive polymer materials.
2. The occurrence and development of GI CSCs have been proven to be related to various mechanisms; however, the exact mechanism is still uncertain. For example, the specific process and influencing factors of the transformation between CSCs and differentiated tumor cells remain unknown. In addition, although some drugs have been proved to have a specific killing effect on CSCs, their mechanism of action is still unclear. This makes the process of targeting CSCs fraught with uncertainty. Clarifying the relationship between CSCs and tumors, drugs and CSCs will provide more ideas for cancer treatment and lay a foundation for the transformation of drugs.
3. In recent studies, the active targeting of CSCs is mostly achieved by coupling a ligand on the surface of nanoparticles. However, multiple specific markers for CSCs have been explored, such as CD133 and CD44. Therefore, researchers may try to attach multiple ligands to a nanoparticle to achieve “multiplex” targeting. In turn, the targeting “precision” of PNP is improved.
4. Some drugs have been shown to have specific effects on CSCs; however, their applications are limited because of their poor water solubility and unsatisfactory bioavailability. The researchers loaded it into PNP, utilized the special microenvironment of the tumor area, and relied on passive targeting to achieve drug delivery to the target area, enabling it to achieve better efficacy at a lower dose. However, passive targeting is not as efficient as active targeting to a certain extent. Therefore, while the drug itself has a specific effect, the active targeting ability of the polymer nanocarrier will be given to it, which will further improve the “targeting” efficiency of the drug.
5. During targeting CSCs, cleverly applying external physical factors to achieve a more diverse targeting process is very popular in recent studies. For example, applying MRI to the targeting process to achieve imaging visualization, combining changes in vitro photothermal conditions with the targeting process for more flexible targeting, etc. The addition of these processes makes the application scenarios of PNP broader and more imaginative.
6. The growth and migration of tumor cells are closely related to the tumor microenvironment and tissue ecosystem. Therefore, appropriate tumor models are very crucial for developing PNP drug delivery systems. Patient-derived xenograft models are often used as “avatars” of cancer, which can simulate the genetic characteristics and microenvironment of tumor patients as much as possible. Polymer nanoparticles can precisely target tissues and cells in PDX in a microenvironment-responsive manner. Additionally, as essential preclinical cancer models, PDXs can be used to comprehensively evaluate and compare the efficacy of different delivery systems, advancing the research of various PNPs in cancer diagnosis and treatment [95]. However, the current costs of developing PDX systems are relatively high, requiring further optimization of their development process.

## Conclusion

Because of the heterogeneity, drug resistance, and complex relationship between CSCs and tumor cells, CSCs have become one of the huge obstacles to the radical cure of GI tumors. Cancer treatment methods focusing on CSCs may become an important breakthrough in eradicating malignant tumors. The emergence of nanomaterials provides more possibilities for the targeted therapy of CSCs. By modifying PNP to optimize their properties, the fabrication of multifunctional and intelligent nanoparticles for targeting CSCs has great advantages with great research potential. This paper summarizes the recent research progress of related polymer nanomaterials in targeting GI CSCs and discusses possible future research directions.

## Data Availability

All data generated or analyzed during this study are included in this published article.

## Abbreviations

ALDH: Aldehyde dehydrogenase
ATRA: All-trans retinoic acid
BTiO2: Black TiO2
CAFs: Cancer-associated fibroblasts
CDF: 3,4-Difluorobenzylidene curcumin
CD44v6-GNS-PEG: CD44v6 monoclonal antibody-conjugated PEG-modified GNS nanoprobes
CSCs: Cancer stem cells
DDC: Diethyldithiocarbamate
Dox: Doxorubicin
DS: Disulfiram
DSPE: Di-stearoyl-phosphatidyl-ethanolamine
EMT: Epithelial–mesenchymal transition
EPND: Epirubicin through physical adsorption to form nanodiamond–epirubicin complex
EPR: Enhanced permeability and retention effect
HA: Hyaluronan
HLs: Hybrid liposome particles
Hh: Hedgehog
HA-SMA: Styrene maleic acid
METase: L-methionine-deamino-γ-mercaptomethane lyase
Gli1: Glioma-associated oncogene homolog 1
GNS: Gold nanostars
GI: Gastrointestinal
Mang-NPs: PLGA nanoparticles containing α-mangostin
MET: Methionine
MNPs: Metallacage-loaded nanoparticles
O2LNCs: Oil liquid nanocapsules
PAMAM: Polyamidoamine
PDX: Patient-derived xenografts model
PEG: Polyethylene glycol
PEG-modified LA-SN38: Linoleic acid-modified 7-ethyl-10-hydroxycamptothecin
PLGA: Poly(lactic-co-glycolic acid)
PNP: Polymer nanoparticles
PLPN: Poly (lactide-co-glycolide)-lecithin-PEG
PLI: Poly-L-lysine-graft-imidazole
PCL: Polycaprolactone
RGD-R8: Cys-RRRRRRRR-c(RGDfK)
ROS: Reactive oxygen species
siRNA: Small interfering RNA
SAL: Salinomycin
SMC2: Structural maintenance of chromosomes protein 2
TM4SF1: Transmembrane 4 L six family member 1
TRAIL: Tumor necrosis factor-related apoptosis-inducing ligand
ZEB1: Zinc finger E-box binding homeobox 1
5-Fu: 5-Fluorouracil
